# Supplementary material for: Immunomodulatory RIG-I Agonist SLR14 Confers Protection Against a Range of Highly Pathogenic Negative Sense RNA viruses
Source: bioRxiv. 2026 Oct 9:2026.10.08.757743. Preprint. [Version 1] doi: 10.64898/2026.10.08.757743 (PMC13692271; doi:10.64898/2026.10.08.757743)
Supplement: Supplementary file 1 [file NIHPP2026.10.08.757743v1-supplement-1.pdf]

bioRxiv preprint doi: https://doi.org/10.64898/2026.10.08.757743; this version posted October 10, 2026. The copyright holder for this preprint (which was not certified by peer review) is the author/funder, who has granted bioRxiv a license to display the preprint in perpetuity. It is made available under a CC-BY 4.0 International license.

Supplementary data

Group	Animal ID	Sex	SLR14 dose (µg)	Treatment Route	FNα concentration (pg/ml)
1	630	M	25	IV	18,331
1	636	M	25	IV	17,453
1	641	F	25	IV	17,555
1	642	F	25	IV	15,851
2	575	M	25	IP	3,736
2	633	M	25	IP	3,531
2	644	F	25	IP	3,759
2	645	F	25	IP	3,763
3	632	M	50	IP	14,327
3	637	M	50	IP	16,750
3	640	F	50	IP	11,632
3	643	F	50	IP	16,436
4	634	M	vehicle	IP	<2.38
4	635	M	vehicle	IP	<2.38
4	638	F	vehicle	IP	<2.38
4	639	F	vehicle	IP	<2.38

Table S1. IFNα serum concentrations 6 hours after SLR14 treatment of BALB/c mice. IFNα serum concentrations were quantified by ELISA. Each row represents one animal. Since the measured IFNα concentrations were outside the range of the standard curve, a sigmoidal, 4PL model was fitted to the standard curve data resulting in an R^2 of 0.9934 using GraphPad Prism version 11 for Mac to interpolate the IFNα concentrations.

bioRxiv preprint doi: https://doi.org/10.64898/2026.10.08.757743; this version posted October 10, 2026. The copyright holder for this preprint (which was not certified by peer review) is the author/funder, who has granted bioRxiv a license to display the preprint in perpetuity. It is made available under a CC-BY 4.0 International license.

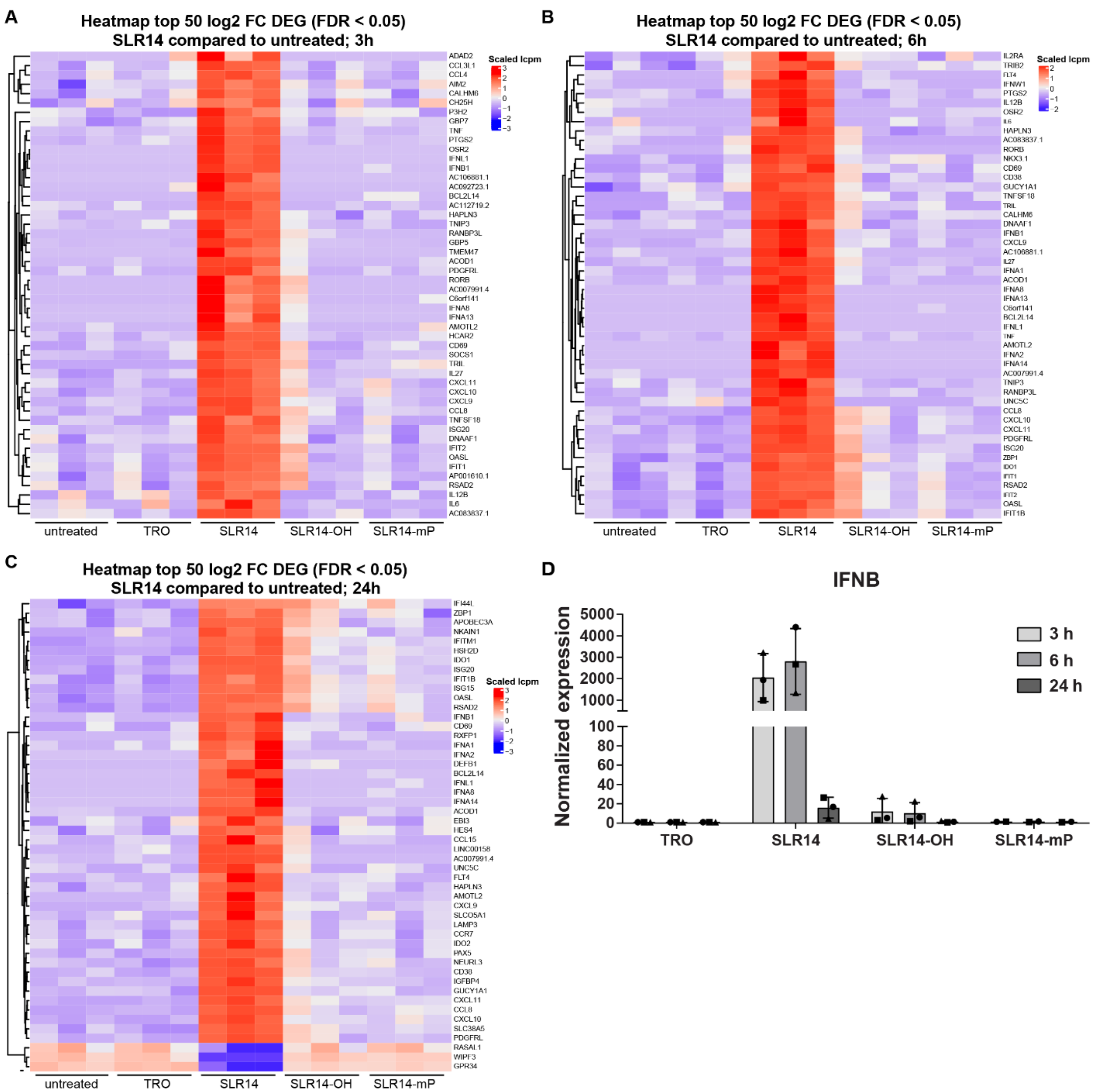

Figure S1. SLR14 treatment of MDMs induces a strong interferon and inflammatory response. MDMs from three different donors seeded in 24-wells were transfected with 1 µg SLR14, 1 µg SLR14-mP, 1 µg SLR14-OH, transfection reagent only (TRO), or left untreated. Total cellular RNA was harvested at the indicated time points for RNA-Seq analysis. (**A-C**) Heatmap of top 50 log2 FC genes of SLR14-treated MDMs compared to untreated cells at **(A)** 3

bioRxiv preprint doi: https://doi.org/10.64898/2026.10.08.757743; this version posted October 10, 2026. The copyright holder for this preprint (which was not certified by peer review) is the author/funder, who has granted bioRxiv a license to display the preprint in perpetuity. It is made available under a CC-BY 4.0 International license.

hours, **(B)** 6 hours, and **(C)** 24 hours. Replicates represent the three donors. **(D)** Quantification of IFNB RNA by RT-qPCR over time. Beta-2-microglobulin primers were used for normalization.

bioRxiv preprint doi: https://doi.org/10.64898/2026.10.08.757743; this version posted October 10, 2026. The copyright holder for this preprint (which was not certified by peer review) is the author/funder, who has granted bioRxiv a license to display the preprint in perpetuity. It is made available under a CC-BY 4.0 International license.

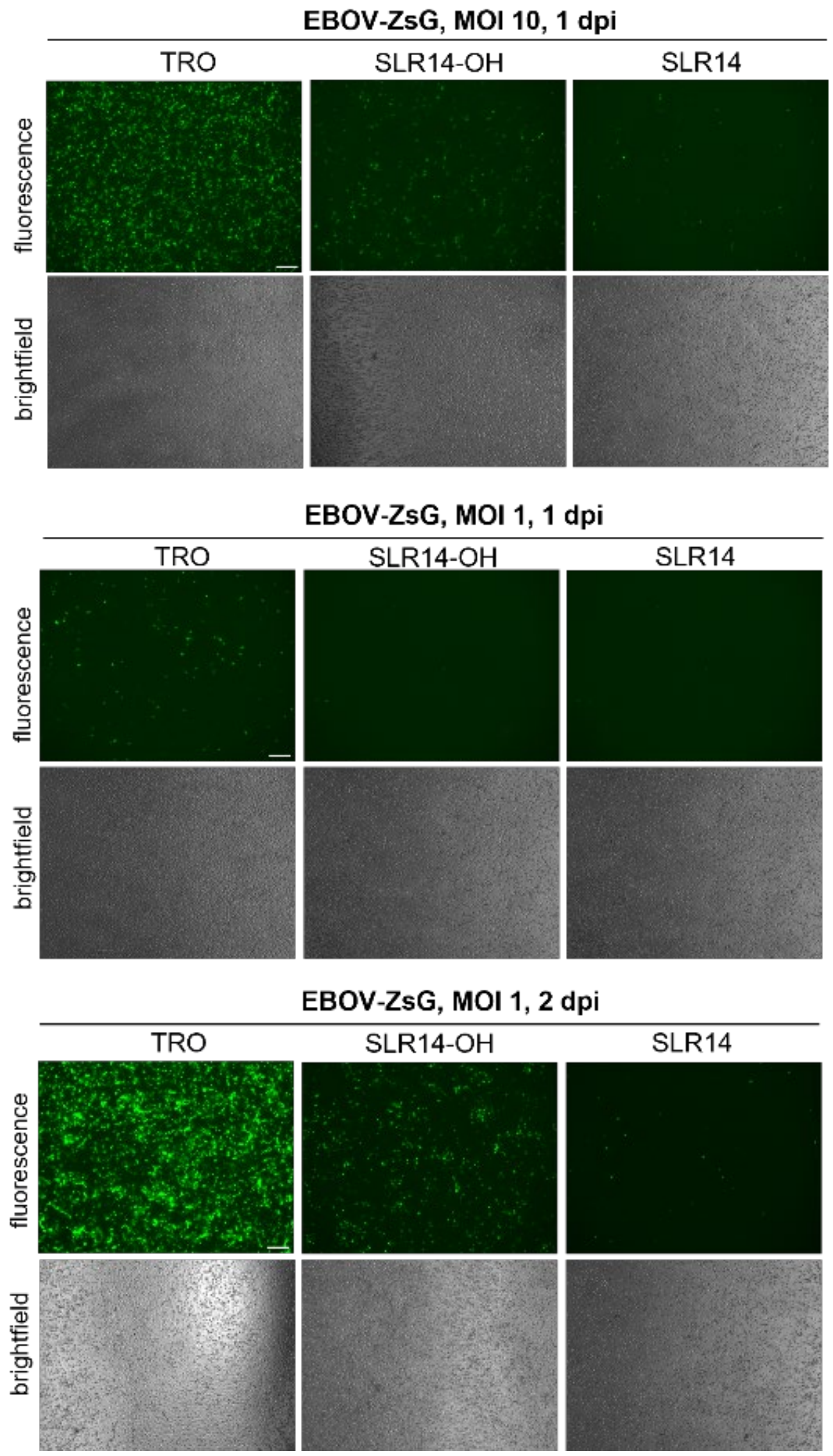

Figure S2. Prophylactic treatment of MDMs with SLR14 inhibits EBOV-ZsGreen infection. MDMs from three different donors seeded in 24-wells ($3x10^5$/well) were transfected with 1 µg SLR14, 1 µg SLR14-mP, 1 µg SLR14-OH, transfection reagent only (TRO), or left untreated. 6 hours post-transfection, cells were infected with recombinant EBOV expressing ZsGreen (EBOV-ZsG) at MOI 1 or 10 as indicated and imaged at 1 and 2 dpi. Representative images are shown. Scale bars = 250 µm.

bioRxiv preprint doi: https://doi.org/10.64898/2026.10.08.757743; this version posted October 10, 2026. The copyright holder for this preprint (which was not certified by peer review) is the author/funder, who has granted bioRxiv a license to display the preprint in perpetuity. It is made available under a CC-BY 4.0 International license.

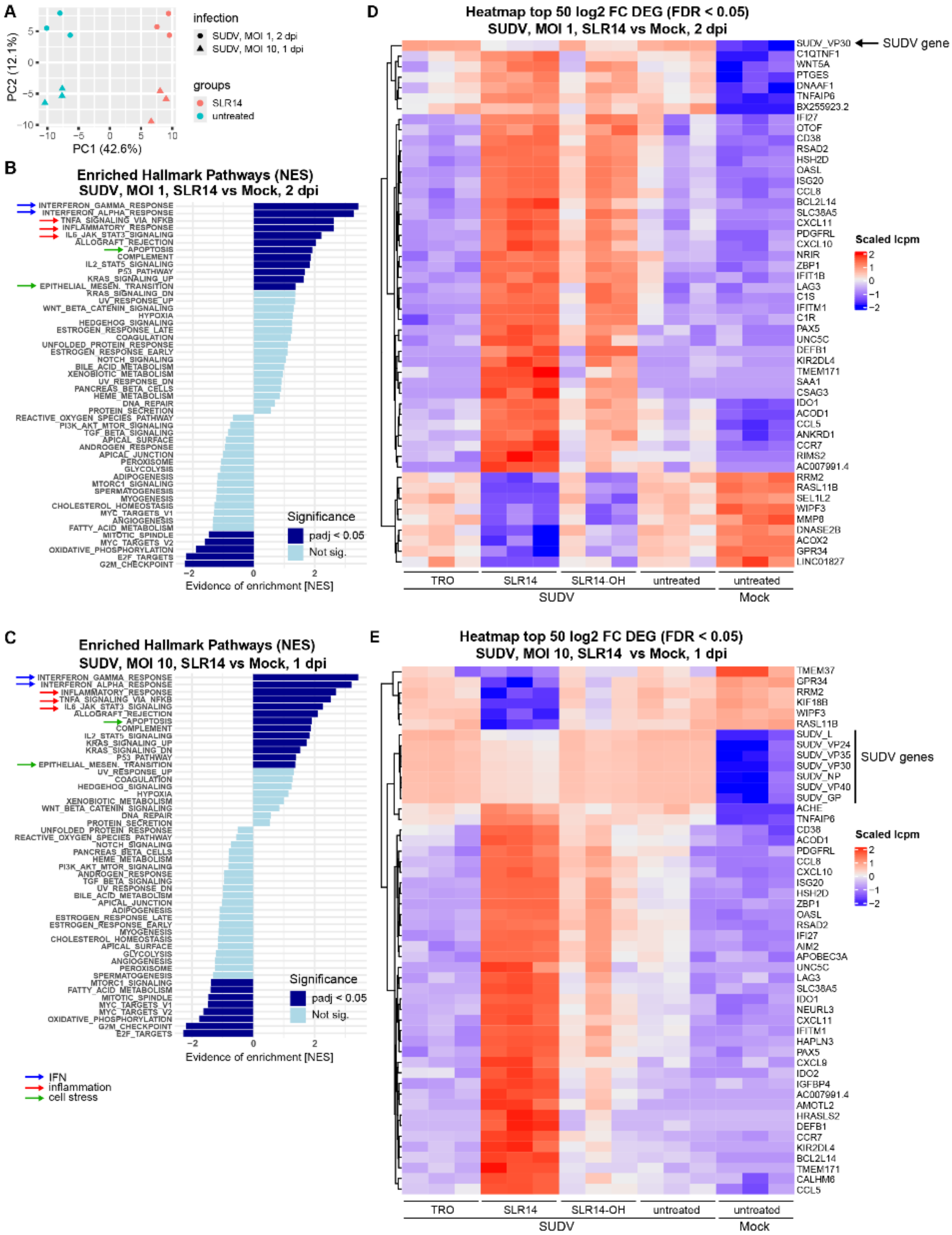

bioRxiv preprint doi: https://doi.org/10.64898/2026.10.08.757743; this version posted October 10, 2026. The copyright holder for this preprint (which was not certified by peer review) is the author/funder, who has granted bioRxiv a license to display the preprint in perpetuity. It is made available under a CC-BY 4.0 International license.

Figure S3. SLR14 treatment induces IFN and inflammatory responses in SUDV-infected MDMs. MDMs from three different donors seeded in 24-wells (3x10^5/well) were transfected with 1 µg SLR14, 1 µg SLR14-OH, transfection reagent only (TRO), or left untreated. 6 hours post-transfection, the cells were infected with SUDV at MOI 1 or MOI 10 and harvested at 1 or 2 dpi. Total cellular RNA was isolated and used for bulk RNA-Seq analysis. (**A**) PCA of the transcriptomic response of the t respective treatments. Different SUDV MOI marked by symbols, different treatment conditions marked by colors. Each sample generated in 3 donors. (**B, C**) GSEA using the Hallmark gene sets on the top DEG sets in SLR14-treated, SUDV-infected MDMs compared to untreated, mock-infected controls. **(B)** SUDV MOI 1, 2d; **(C)** SUDV MOI 10, 1d. Light blue bars, not significant; dark blue bars, statistically significant ($p < 0.05$). (**D, E**) Heatmap of top 50 log2 FC genes in SLR14-treated, SUDV-infected MDMs compared to mock-infected controls for all tested treatment conditions. Replicates represent the three donors. **(D)** SUDV MOI 1, 2d; **(E)** SUDV MOI 10, 1d.

bioRxiv preprint doi: https://doi.org/10.64898/2026.10.08.757743; this version posted October 10, 2026. The copyright holder for this preprint (which was not certified by peer review) is the author/funder, who has granted bioRxiv a license to display the preprint in perpetuity. It is made available under a CC-BY 4.0 International license.

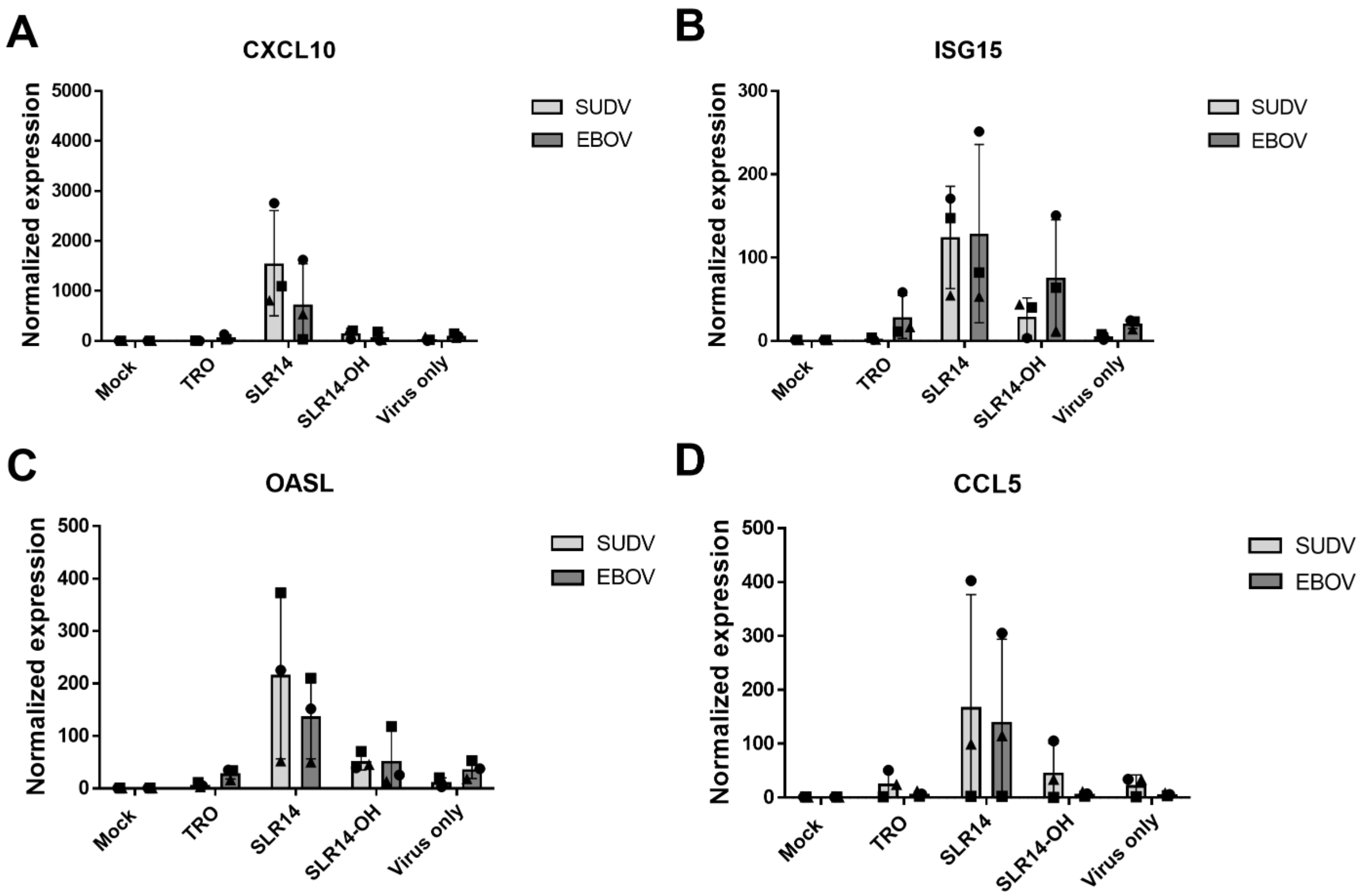

Figure S4. ISG and chemokine expression in SLR14-treated and EBOV or SUDV infected MDMs. MDMs from three different donors seeded in 24-wells (3x10^5/well) were transfected with 1 µg SLR14, 1 µg SLR14-OH, transfection reagent only (TRO), or left untreated. 6 hours post-transfection, the cells were infected with EBOV or SUDV at MOI 1 and harvested at 2 dpi. Total cellular RNA was isolated at indicated time points. Quantification of **(A)** CXCL10, **(B)** ISG15, **(C)** OASL, **(D)** CCL5 RNA by RT-qPCR. Beta-2-microglobulin primers were used for normalization. Data presented as fold expression relative to mock control. Symbols indicate different donors.

bioRxiv preprint doi: https://doi.org/10.64898/2026.10.08.757743; this version posted October 10, 2026. The copyright holder for this preprint (which was not certified by peer review) is the author/funder, who has granted bioRxiv a license to display the preprint in perpetuity. It is made available under a CC-BY 4.0 International license.

A

Gene	log2FoldChange	pvalue
CXCL11	15.51595922	2.50E-68
CXCL10	15.41624815	2.23E-41
ART3	14.56610951	8.91E-107
CXCL9	14.11247773	3.11E-47
IDO1	12.86417465	4.89E-121
LGALS17A	12.12934666	1.81E-30
ACOD1	11.51189605	8.32E-70
CCL8	11.48316097	4.25E-103
IFNB1	10.73993457	4.51E-39
IFNL1	10.36774402	1.38E-36
IFNA8	10.00235908	2.20E-26
IGFBP4	9.98212373	5.24E-179
CCR7	9.907713079	2.38E-109
IFNA14	9.894960039	2.04E-22
IFNA2	9.817038312	6.50E-16
CSAG3	9.787032136	1.14E-53
HAPLN3	9.692090278	5.88E-170
CSAG2	9.631636834	1.13E-47
SLC38A5	9.345445432	5.33E-134
NEURL3	9.285449436	2.85E-166
DEFB1	9.207466265	3.07E-24
RSAD2	9.004069167	1.03E-37
CD38	8.887697464	2.43E-101
TRIL	8.886248664	1.22E-40
ISG20	8.876261097	5.37E-246
ZBP1	8.851503883	6.08E-45
IFNA13	8.775762655	6.15E-30
TOMM20P2	8.75969545	4.82E-39
IFNW1	8.753558585	6.50E-22
OASL	8.678733775	2.06E-47
AMOTL2	8.603597467	1.69E-29
NKX3-1	8.564565392	5.18E-28
IFNA10	8.508103781	1.99E-11
UNC5C-AS1	8.314406608	3.14E-27
IFNA7	8.25957546	1.92E-19
IFNA1	8.232161956	2.97E-19
PAX5	8.210263629	4.04E-65
RN7SL124P	8.08857282	3.01E-35
CD69	7.957521493	6.09E-55
EBI3	7.91839979	7.76E-46
OR2I1P	7.907069294	1.18E-23
CCL5	7.892703157	6.09E-154
TAF5LP1	7.878735869	2.19E-52

B

Gene
IFNA1
IFNA2
IFNA7
IFNA8
IFNA10
IFNA13
IFNA14
IFNA21
IFNW1
NKX3-1
TRIL

C

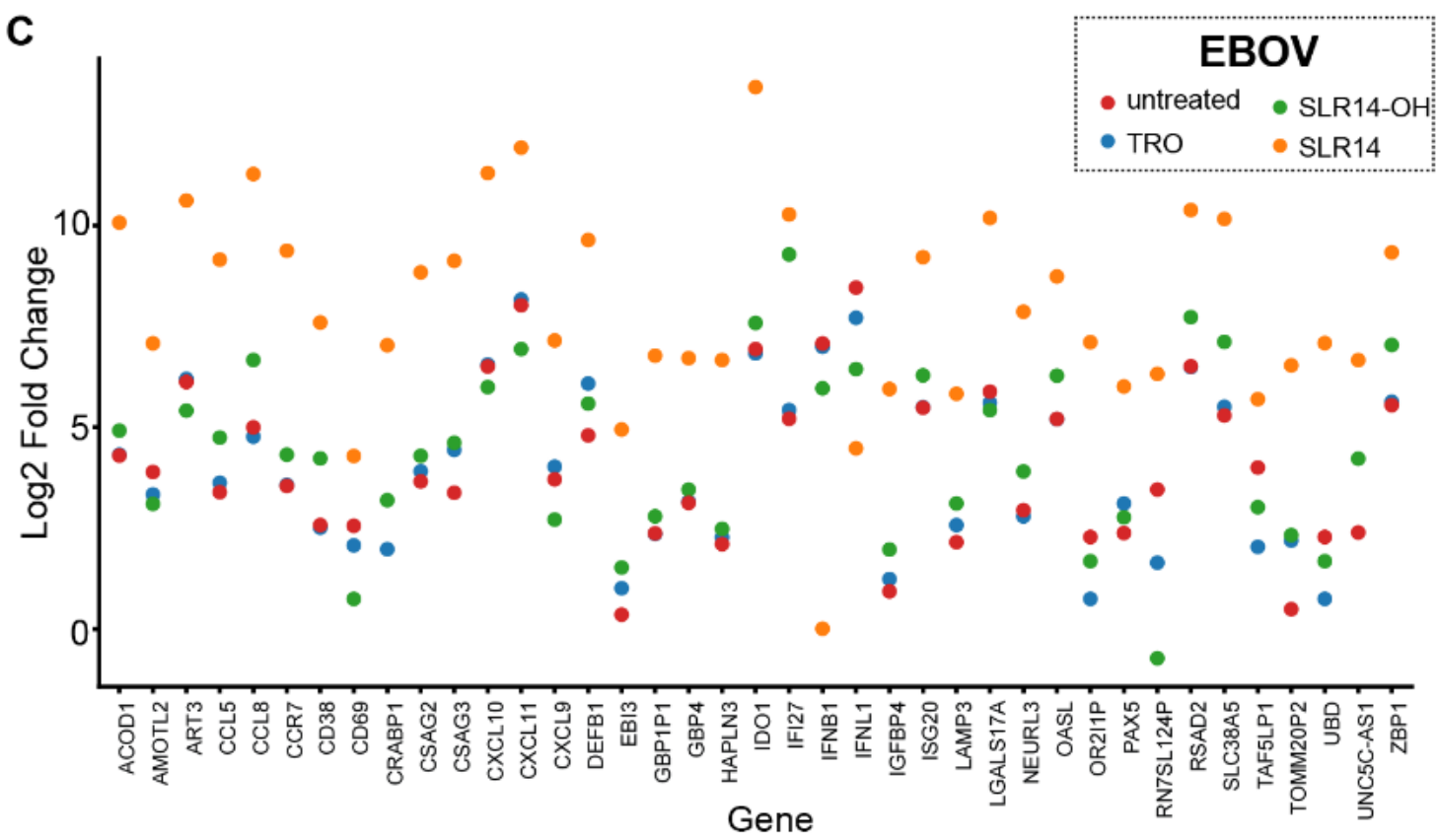

D

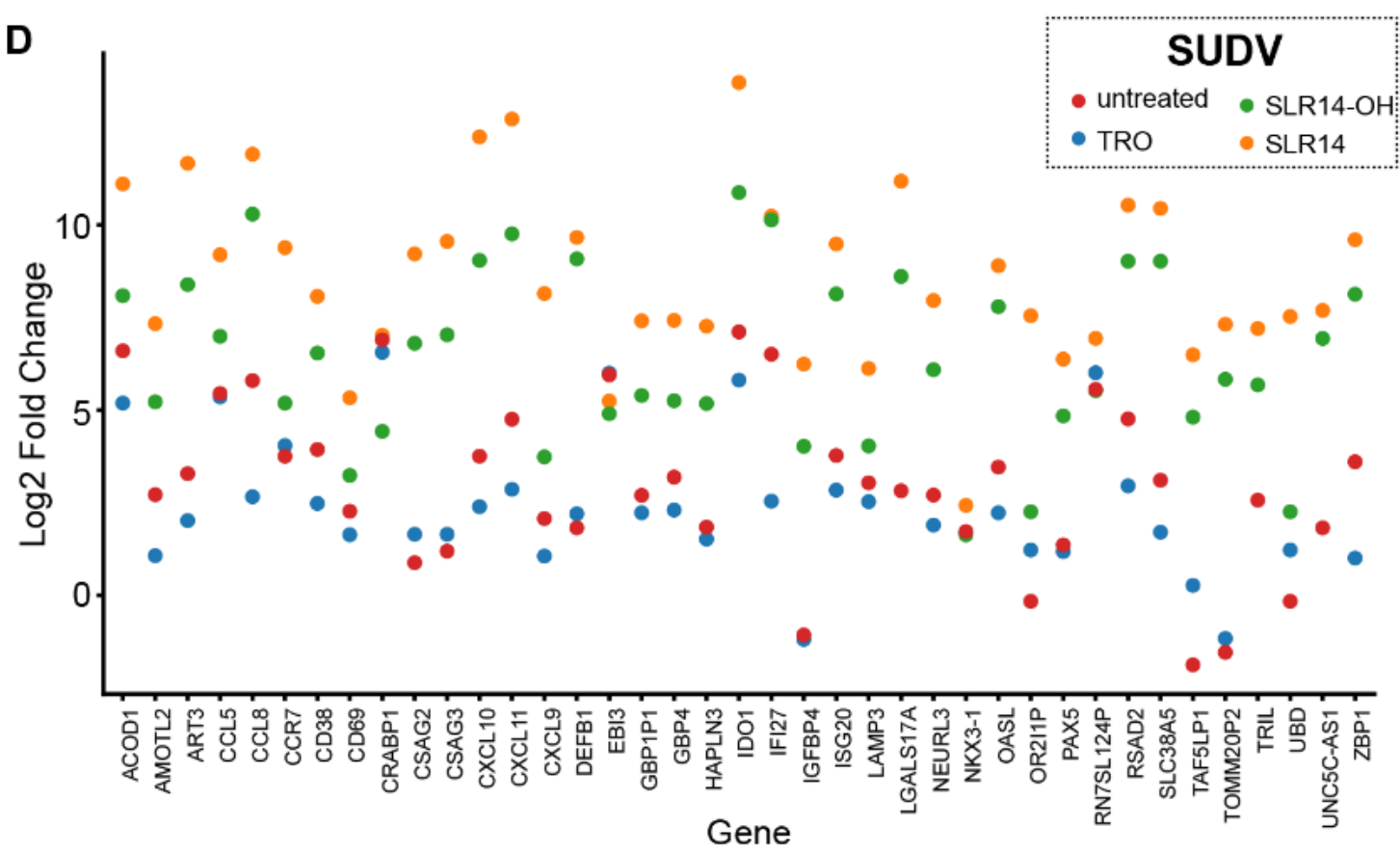

Fig. S5. Supplemental figure to figure 4. (**A**) List of the 50 most highly upregulated genes in SLR14-treated, uninfected MDMs at 24 hours post-treatment. (**B**) List of the 11 genes that were exclusively upregulated in SLR14-treated, uninfected MDMs at 24 hours post-treatment. (**C and D**) Comparison of the 39 shared genes upregulated by SLR14 treatment in uninfected cells to the same set of genes in (**C**) EBOV and (**D**) SUDV infection under various treatment conditions. Treatment conditions are visualized by different colors.

bioRxiv preprint doi: https://doi.org/10.64898/2026.10.08.757743; this version posted October 10, 2026. The copyright holder for this preprint (which was not certified by peer review) is the author/funder, who has granted bioRxiv a license to display the preprint in perpetuity. It is made available under a CC-BY 4.0 International license.

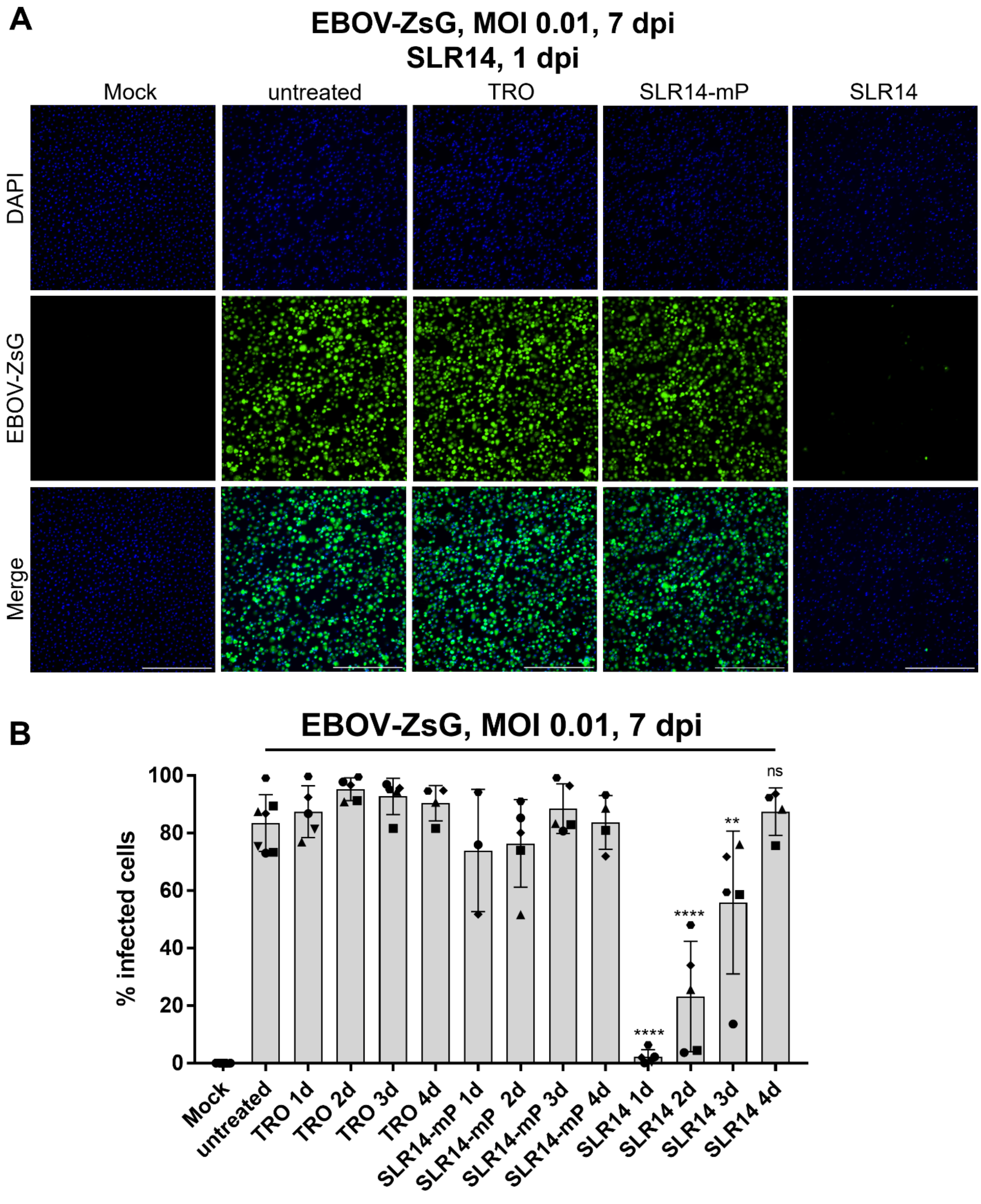

Figure S6. SLR14 blocks EBOV-ZsGreen infection of MDMs when administered post-infection. MDMs obtained from 3 to 5 donors seeded in 24-well plates (3x10^5/well) were mock-

bioRxiv preprint doi: https://doi.org/10.64898/2026.10.08.757743; this version posted October 10, 2026. The copyright holder for this preprint (which was not certified by peer review) is the author/funder, who has granted bioRxiv a license to display the preprint in perpetuity. It is made available under a CC-BY 4.0 International license.

infected or infected with EBOV-ZsG at MOI 0.01. At 1 to 4 dpi, the cells were transfected with 1 µg SLR14, 1 µg SLR14-mP or transfection reagent only (TRO), or left untreated. Cells were imaged at 7 dpi. (**A**) Representative images of samples treated at 1d with SLR14. Scale bars = 500 µm. (**B**) EBOV-ZsG infection rates as quantified from multiple images ($n \geq 2$ per donor) of MDMs using QuPath. Donors represented by different symbols. Significant differences between conditions compared to untreated control were determined by unpaired two-tailed T-test, P-values: *<0.1; **<0.01; ***<0.001; ****<0.0001; ns: not significant.

bioRxiv preprint doi: https://doi.org/10.64898/2026.10.08.757743; this version posted October 10, 2026. The copyright holder for this preprint (which was not certified by peer review) is the author/funder, who has granted bioRxiv a license to display the preprint in perpetuity. It is made available under a CC-BY 4.0 International license.

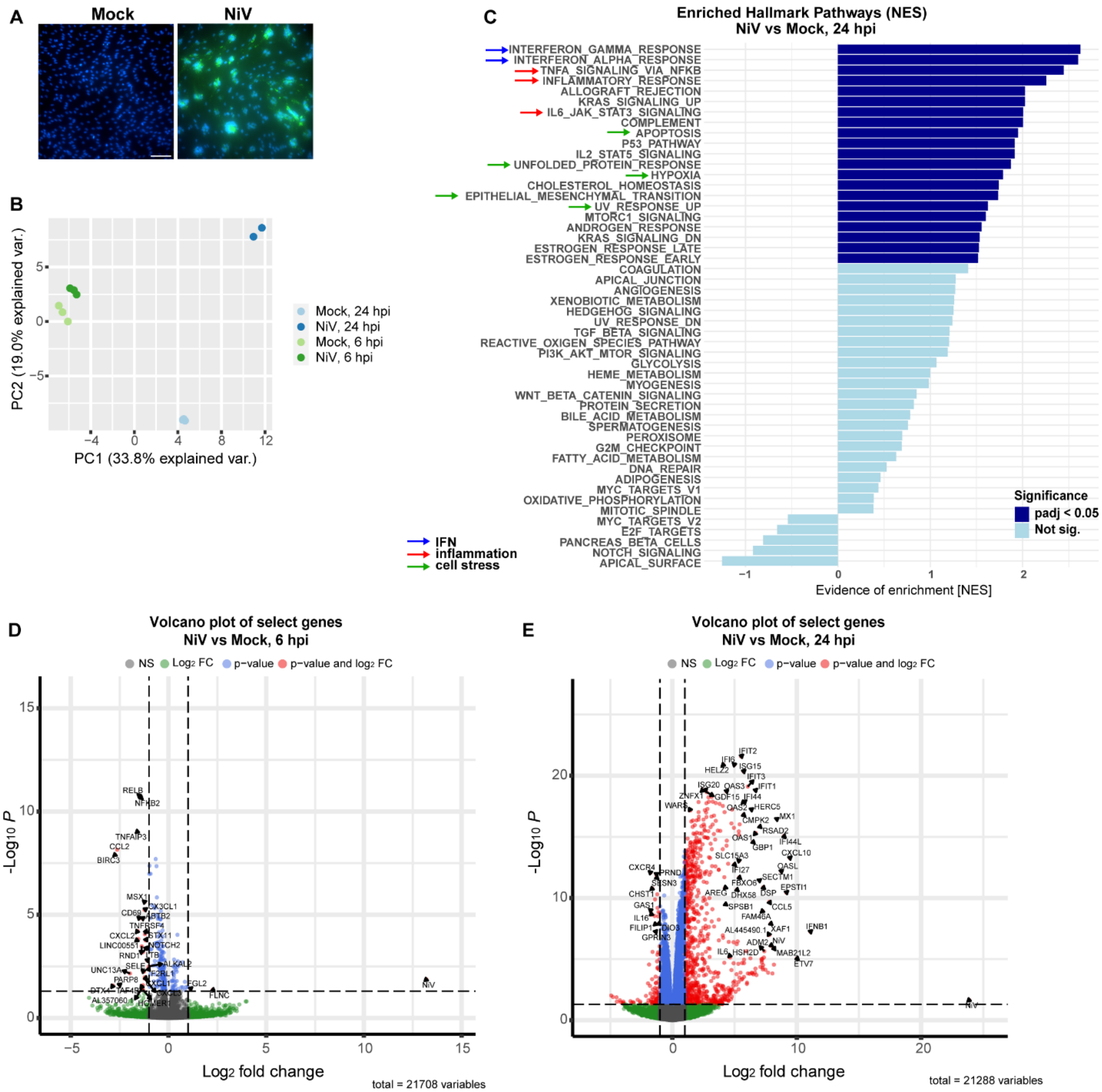

Figure S7. Inflammatory responses in iECs infected with NiV (A) $1.5x10^5$ iECs seeded per well of 8-well chamber slides were infected with NiV at and MOI of 10. Scale bar = 100 µm **(B)** iEC seeded per well of 24-well plates were infected with NiV Bangladesh at MOI 10. The cells were harvested at the indicated time points and subjected to RNA-Seq. PCA of the transcriptomic response of the respective treatments. Each sample generated in triplicate except NiV, 24 h which was done in duplicate. **(C)** GSEA using the Hallmark gene sets on the top DEG sets in NIV-

bioRxiv preprint doi: https://doi.org/10.64898/2026.10.08.757743; this version posted October 10, 2026. The copyright holder for this preprint (which was not certified by peer review) is the author/funder, who has granted bioRxiv a license to display the preprint in perpetuity. It is made available under a CC-BY 4.0 International license.

infected iECs compared to untreated, mock-infected controls at 24 hpi. **(D, E)** Volcano plot of DEGs in NiV-infected MDMs compared to mock-infected controls. Select IFNs, ISGs, and viral genes are labeled. **(D)** NIV, 6 hpi. **(E)** NIV, 24 hpi.

bioRxiv preprint doi: https://doi.org/10.64898/2026.10.08.757743; this version posted October 10, 2026. The copyright holder for this preprint (which was not certified by peer review) is the author/funder, who has granted bioRxiv a license to display the preprint in perpetuity. It is made available under a CC-BY 4.0 International license.

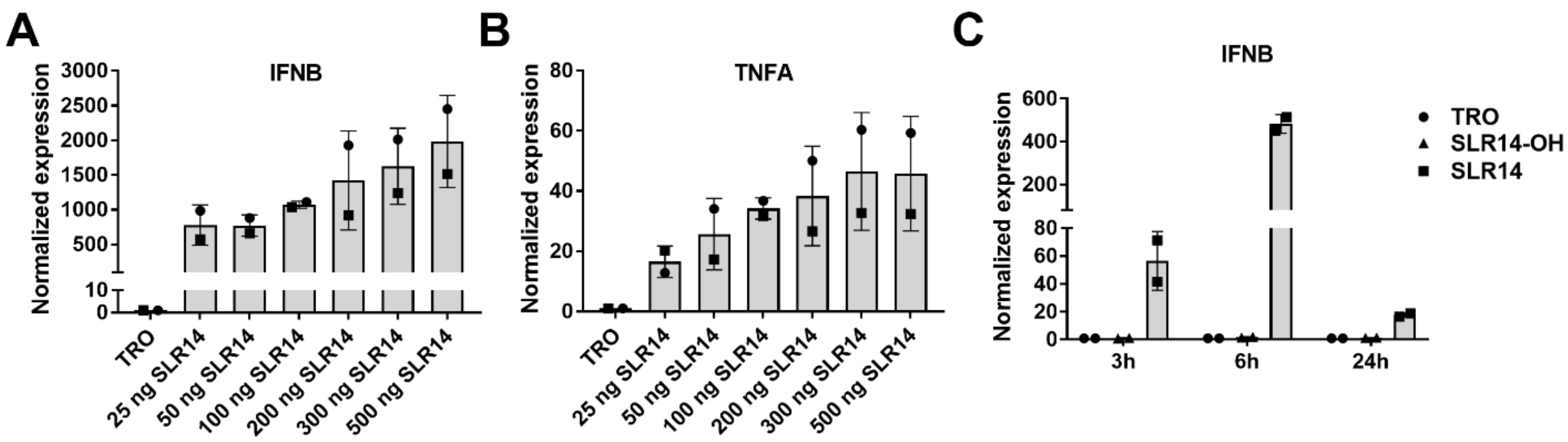

Figure S8. Inflammatory responses in iECs infected treated with SLR14. (**A and B**) iECs seeded in 24-well plates (1.8x10^{5}/well) were transfected with increasing amounts of SLR14 or transfection reagent only (TRO) as indicated. 6 hours post-transfection, total RNA was harvested and used for RT-qPCR using primers binding to (**A**) IFNB1 or (**B**) TNFA mRNA. Normalization of RT-qPCR data was performed using beta-microglobulin as the reference gene. (**C**) iECs were transfected with 25 ng SLR14, 25 ng SLR14-OH, or TRO. Total cellular RNA was harvested at the indicated time points and subjected to RT-qPCR analysis using IFNB1-specific primers. Data presented as fold expression relative to TRO control. Different symbols represent two biological replicates.
